# 厄洛替尼治疗34例肺鳞癌患者的疗效观察

**DOI:** 10.3779/j.issn.1009-3419.2016.10.08

**Published:** 2016-10-20

**Authors:** 芳 程, 静 王, 洁 陈, 镨元 邢, 峻岭 李

**Affiliations:** 1 100021 北京，国家癌症中心/中国医学科学院北京协和医学院肿瘤医院 National Cancer Center/Cancer Hospital, Chinese Academy of Medical Sciences and Peking Union Medical College, Beijing 100021, China; 2 100122 北京，北京市朝阳区三环肿瘤医院 Beijing Chaoyang Sanhuan Cancer Hospital, Beijing 100122, China

**Keywords:** 肺鳞癌, 厄洛替尼, EGFR-TKI, Lung neoplasms, Erlotinib, EGFR-TKI

## Abstract

**背景与目的:**

表皮生长因子受体酪氨酸激酶抑制剂（epidermal growth factor receptor tyrosine kinase inhinitor, EGFR-TKI）通过影响肿瘤的信号传导来抑制肿瘤发展，它具有良好的安全性。本研究旨在观察厄洛替尼治疗肺鳞癌患者的基本情况及生存时间。

**方法:**

对34例使用厄洛替尼靶向治疗的肺鳞癌患者，给予厄洛替尼150 mg每日1次口服直至病情进展或因不良反应不能耐受为止。

**结果:**

截至2016年6月13日，34例患者中一线治疗患者7例，维持治疗患者6例（其中1例因严重毒性反应停药），二线治疗患者9例（其中1例患者后续再次使用厄洛替尼），三线治疗患者5例，三线以上治疗患者7例（其中1例患者为2次使用厄洛替尼）；确认死亡患者11例。除去1例严重不良反应的患者，口服厄洛替尼后最短疾病无进展生存时间（progression-free survival, PFS）为1个月，最长PFS为55个月，中位PFS为3.5个月。

**结论:**

对于一部分晚期不能耐受化疗或拒绝化疗，并且基因状况不明的肺鳞癌患者来说，厄洛替尼有一定疗效，绝大部分患者不良反应可耐受。

## Abstract (en)

**Background and objective:**

Epidermal growth factor receptor tyrosine kinase inhinitors (EGFR-TKIs) inhibit tumor growth by affecting signal transduction, and are well tolerated. The aims of this study was to observe the effect of erlotinib in patients with squamous cell lung cancer.

**Methods:**

The 34 patients with squamous cell lung cancer treated with erlotinib 150 mg orally once daily until disease progression or intolerable adverse reactions.

**Results:**

The 7 patients were treated with erlotinib as first-line treatment, 6 patients as maintenance therapy (1 case withdrawal of severe toxicity), 9 patients second-line treatment, 5 patients as third-line and 7 patients as further-line therapy. 11 patients died. The median progression-free survival (PFS) was 3.5 months, ranging from 1 month to 55 months (the patient withdrawal of severe toxicity was not included).

**Conclusion:**

Erlotinib was effective for patients with squamous cell lung cancer who can not tolerate chemotherapy or refused chemotherapy and with unknown EGFR status. Adverse reactions were tolerable.

晚期非小细胞肺癌（non-small cell lung cancer, NSCLC）是目前全世界死亡率最高的恶性肿瘤，大多数患者诊断时已是晚期，通常不能治愈。对于表皮生长因子受体（epidermal growth factor receptor, *EGFR*）基因突变状态为野生型的患者，第三代化疗药物联合铂类的化疗方案虽然是一线治疗的标准选择，但疗效已达到平台。化疗对晚期或复发的NSCLC最好的有效率在20%-40%，中位生存时间仅8个月-10个月，1年生存率为30%-40%。化疗仅对于部分晚期或复发的NSCLC患者起到了延长生存和改善生活质量的作用。

近几年，随着分子生物学技术的进展和人类对癌症发病机制的认识，表皮生长因子受体酪氨酸激酶抑制剂（EGFR tyrosine kinase inhinitor, EGFR-TKI）治疗肿瘤便是抑制肿瘤生长的一种新的治疗方法。EGFR-TKI治疗通过影响肿瘤的信号传导来抑制肿瘤发展，它除了具有选择性-亚裔、腺癌、非吸烟患者、*EGFR*突变者疗效优于化疗；还具有良好的安全性，并且使用方便，能够维持较好的生活质量。目前已有大量研究证明*EGFR*基因突变的晚期NSCLC患者，厄洛替尼一线治疗疗效显著优于化疗。对于敏感突变人群，厄洛替尼的获益贯穿于NSCLC患者所有组织学类型、吸烟状态、年龄及性别^[1, 2]^。

## 1 资料与方法

### 1.1 病例资料

收集我科2011年以前30例基因突变状况不明及4例*EGFR* 19或21外显子突变口服厄洛替尼的肺鳞癌/腺鳞癌患者，并随访至2016年6月13日。其中男性23例，女性11例，年龄范围40岁-78岁，65岁以下患者20例，65岁以上患者14例。鳞癌26例，腺鳞癌7例，癌1例；吸烟者16例，非吸烟者18例；其中一线治疗患者7例，维持治疗患者6例（其中1例因严重毒性反应停药），二线治疗患者9例（其中1例患者后续再次使用厄洛替尼），三线治疗患者5例，三线以上治疗患者7例（其中1例患者为2次使用厄洛替尼）。死亡的11例患者中，生存时间最短5.2个月，最长35.7个月。

### 1.2 诊断依据

该34例患者均由病理证实为肺癌，其中鳞癌26例，腺鳞癌7例，癌1例。

### 1.3 治疗方法

厄洛替尼150 mg/次，口服，每天1次，直到病情进展或因不良反应不能耐受为止。

### 1.4 随访

所有患者在首次服药1个月后确定疗效，之后每1个月-2个月复查1次，复查内容包括胸腹计算机断层扫描（computed tomography, CT）、肺系统肿瘤标志物及血生化检查；临床考虑有转移的患者加做相应部位的影像学检查（包括脑核磁、骨扫描、颈部CT等）。部分病情进展停止服药的患者仍然继续接受随访，直至随访到死亡时间。

### 1.5 统计学方法

采用SPSS 17.0统计软件进行统计处理，用*Kaplan*-*Meier*进行生存分析，使用*Log*-*rank*检验，*P*＜0.05为差异有统计学意义。

## 2 结果

34例患者中位无进展生存期（progression-free survival, PFS）3.5个月。其中男性23例，中位PFS 3.0个月，女性11例，中位PFS 3.6个月，无统计学差异（*P*=0.8）。65岁以下患者20例，中位PFS 3.5个月，≥65岁患者14例，中位PFS 3.0个月，无统计学差异（*P*=0.2）。鳞癌26例，腺鳞癌7例，癌1例；吸烟者16例，中位PFS 3.0个月，非吸烟者18例，中位PFS 7.5个月，虽然非吸烟患者中位PFS较吸烟患者长4.5个月，但无统计学差异（*P*=0.6）；其中一线治疗患者7例，中位PFS 3.6个月，维持治疗患者6例（其中1例因严重毒性反应停药），中位PFS 3.0个月，二线治疗患者9例（其中1例患者后续再次使用厄洛替尼），中位PFS 6.5个月，三线治疗及以上患者12例（其中1例患者为2次使用厄洛替尼），中位PFS 2.1个月（*P*=0.78）（表 1）。除去1例严重不良反应的患者，33例口服厄洛替尼的患者最短疾病无进展时间1个月，最长疾病无进展时间55个月，中位PFS 3.5个月。在随访至死亡的11例患者中，生存时间最短5.2个月，最长35.7个月，中位总生存19.1个月。

**1:** 34例患者的基线特征 34 patients baseline characteristics

Characteristics	No. of patients [*n*(%)]	Progression-free survival (PFS)(months)	*P*
Gender			
Male	23 (67.6)	3.0	0.8
Female	11 (32.4)	3.6	
Age			
Range	40-78		
＜65 years	20 (58.9)	3.5	0.2
≥65 years	14 (41.1)	3.0	
Smoking			
Never	18 (52.9)	7.5	0.6
Ever	16 (47.1)	3.0	
Histological type			
Squamous cell	26 (76.5)	2.1	0.2
Adenosquamous carcinoma	7 (20.6)	8.7	
Carcinoma	1 (2.9)		
Therapy			
1^st^ line	7 (20.6)	3.6	0.8
Maintain	6 (17.6)	3.0	
2^nd^ line	9 (26.5)	6.5	
3^rd^ or further line	12 (35.3)	2.1	

## 3 讨论

厄洛替尼是小分子口服EGFR-TKI，与另一种EGFR-TKI吉非替尼不同的是，厄洛替尼与靶点的亲和力强、特异性高、血浆浓度高，半衰期短，口服厄洛替尼较吉非替尼易于吸收，对于脑转移患者来说有一定疗效^[3]^。2006年美国临床肿瘤学会（American Society of Clinical Oncology, ASCO）公布显示，厄洛替尼用于吸烟的男性鳞癌患者和肿瘤患者能显著改善这类患者的生存期^[4-7]^。2010年以前厄洛替尼主要使用在两个或者两个以上化疗方案失败，卡氏功能状态评分（Karnofsky performance status, KPS）0分-1分的局部晚期或者转移性NSCLC患者，当时尚未提及特定人群的基因检测情况，但是一部分患者在发现NSCLC后立即使用厄洛替尼治疗，效果很好。直至2010年美国国立综合癌症网络（National Comprehensive Cancer Network, NCCN）指南提及厄洛替尼可用于NSCLC *EGFR*突变人群的一线治疗。

目前的研究大部分都是对厄洛替尼治疗NSCLC的疗效分析，不论肿瘤分期如何都可以选择厄洛替尼，*EGFR*突变的患者有效率较高，临床发现对于突变状况不明的患者，厄洛替尼也有疗效。晚期多程治疗后的患者，体质较差，耐受性欠佳，使用厄洛替尼有着明显的优势，能够在较少的毒副反应、安全系数高的前提下，最大限度延长患者生存时间，改善生活质量，为下一步治疗争取机会^[8-10]^。

所以，对于晚期NSCLC患者来说，治疗应尽可能地依据循证医学的证据以及批准的适应症，避免或减少对患者的伤害，根据患者意愿满足患者要求。对于一部分晚期不能耐受化疗或拒绝化疗，并且基因状况不明的肺鳞癌患者来说，为争取快速控制症状和疾病进展，避免不能耐受的毒性，厄洛替尼似乎亦是可以考虑的选择。
